# Relationship between Changes in Myocardial F-18 Fluorodeoxyglucose Uptake and Radiation Dose after Adjuvant Three-Dimensional Conformal Radiotherapy in Patients with Breast Cancer

**DOI:** 10.3390/jcm9030666

**Published:** 2020-03-02

**Authors:** In Young Jo, Jeong Won Lee, Woo Chul Kim, Chul Kee Min, Eun Seog Kim, Seung-Gu Yeo, Sang Mi Lee

**Affiliations:** 1 Department of Radiation Oncology, Soonchunhyang University Cheonan Hospital, 31 Suncheonhyang 6-gil, Dongnam-gu, Cheonan, Chungcheongnam-do 31151, Korea; 2 Department of Nuclear Medicine, Catholic Kwandong University College of Medicine, International St. Mary’s Hospital, Simgok-ro 100-gil 25, Seo-gu, Incheon 22711, Korea; 3 Department of Radiation Oncology, Soonchunhyang University College of Medicine, Soonchunhyang University Cheonan Hospital, 31 Suncheonhyang 6-gil, Dongnam-gu, Cheonan, Chungcheongnam-do 31151, Korea; 4 Department of Nuclear Medicine, Soonchunhyang University Cheonan Hospital, 31 Suncheonhyang 6-gil, Dongnam-gu, Cheonan, Chungcheongnam-do 31151, Korea

**Keywords:** breast cancer, fluorodeoxyglucose F-18, positron emission tomography, radiotherapy, cardiomyopathy, radiation toxicity

## Abstract

This study aimed to assess the relationship between radiation dose and changes in the irradiated myocardial F-18 fluorodeoxyglucose (FDG) uptake after radiotherapy (RT) in breast cancer patients. The data of 55 patients with left and 48 patients with right breast cancer who underwent curative surgical resection and adjuvant three-dimensional conformal RT and staging (PET1), post-adjuvant chemotherapy (PET2), post-RT (PET3), and surveillance (PET4) FDG positron emission tomography/computed tomography (PET/CT) were retrospectively reviewed. The median interval between PET1 and curative surgical resection, between the end of adjuvant chemotherapy and PET2, between the end of RT and PET3, and between the end of RT and PET4 were five days, 13 days, 132 days, and 353 days, respectively. The myocardial-to-blood pool uptake ratio was measured in all patients. For patients with left breast cancer, the 30 Gy- (30 Gy) and 47.5 Gy-irradiated myocardium-to-low-irradiated myocardium (47.5 Gy) FDG uptake ratios were additionally measured. There were no differences in the myocardial-to-blood pool uptake ratios between left and right breast cancer on all PET scans. For left breast cancer, higher 30 Gy and 47.5 Gy uptake ratios were observed on PET3 than on PET1 and PET2. Both uptake ratios decreased on PET4 compared to PET3, but, were still higher compared to PET1. On PET3 and PET4, the 47.5 Gy were higher than the 30 Gy uptake ratios, while there were no differences between them on PET1 and PET2. Although the whole myocardium FDG uptake showed no significant change, the irradiated myocardium FDG uptake significantly increased after RT and was related to radiation dose to the myocardium in breast cancer patients. These results might be an imaging evidence that supports the increased risk of heart disease after RT in patients with left breast cancer.

## 1. Introduction

Breast conserving surgery followed by adjuvant radiotherapy (RT) is widely accepted as an essential part of breast cancer treatment [1,2]. Adjuvant RT reduces the risk of mortality by 16% and that of local recurrence by more than 50% in patients with breast cancer [1,3]. However, for patients with left breast cancer, some territory of the heart is often included in the RT field, which could result in radiation-induced damage to the myocardium and coronary artery [4,5], and previous clinical studies have reported increased risk of ischemic heart disease, myocardial infarction, and cardiac disease-related mortality in patients with left breast cancer compared to those with right breast cancer [6,7,8]. In a previous dosimetry study, patients with left breast cancer had absorbed a dose of 2.3 ± 0.7 Gy to the heart and the left anterior descending coronary artery showed the highest radiation dose (7.6 ± 4.5 Gy) among the coronary arteries, while all patients with right breast cancer had absorbed a dose lower than 2.0 Gy [5]. These results emphasize the need to develop advanced RT techniques that can reduce the radiation dose to the heart and to screen patients with left breast cancer for radiation-induced heart disease [4,9,10]. 

F-18 Fluorodeoxyglucose (FDG) positron emission tomography/computed tomography (PET/CT) has been used for initial staging, treatment response monitoring, and predicting prognosis in patients with breast cancer [11,12,13,14]. As FDG uptake is proportional to glucose metabolism, FDG PET/CT can be also used to estimate the degree of glucose metabolism in normal organ tissue [15,16]. Although increased FDG uptake was observed in the myocardium in ischemic conditions [17], various degrees of myocardial FDG uptake have been reported with oncology FDG PET/CT protocols [18,19], and the clinical significance of FDG uptake in the myocardium of patients with malignant diseases has been long neglected. However, recent studies with patients with lung and esophageal cancer have reported focally increased FDG uptake in the myocardium within the radiation field on post-RT PET/CT images [20,21]. Because high dose radiation exposure can lead to glycolysis upregulation in the myocardium, increased FDG uptake in the myocardium after RT in lung and esophageal cancer is considered to result from radiation-induced myocardial damage, suggesting that myocardial FDG uptake acts as a surrogate marker for myocardial damage [20,21,22]. However, until now, the changes in the FDG uptake pattern in the myocardium have not been assessed in patients with breast cancer undergoing adjuvant RT. Furthermore, most previous studies have evaluated myocardial FDG uptake by visual analysis or by measuring the maximum FDG uptake of certain myocardial portions, and only few have measured and compared myocardial FDG uptake according to dose-distribution maps for RT [20,21,23,24,25].

In the present study, we retrospectively enrolled patients with breast cancer who had undergone both staging and post-RT PET/CT and measured the FDG uptake of the whole and of the irradiated myocardium on both staging and post-RT PET/CT images by fusing FDG PET/CT images with dose-distribution maps on RT simulation CT images. Using these myocardial FDG uptake values, we aimed to investigate whether FDG uptake of the whole and irradiated myocardium changes after adjuvant three-dimensional conformal radiotherapy (3D-CRT) and whether the changes in myocardial FDG uptake are related to the radiation dose to the myocardium in patients with breast cancer.

## 2. Materials and Methods

### 2.1. Study Population

This retrospective study was approved by the Institutional Review Board of our university (IRB number: SCH-2019-11-008), which waived the requirement for informed consent. A total of 393 women who were histopathologically diagnosed with invasive breast cancer and underwent staging FDG PET/CT between February 2012 and December 2016 were screened for study enrollment. Among them, 106 patients who: (1) had no evidence of distant metastasis on staging imaging studies and underwent curative surgical resection, (2) received adjuvant 3D-CRT after curative surgical resection with/without other adjuvant treatment, and (3) underwent post-RT FDG PET/CT; thus, underwent both staging and post-RT PET/CT scans were initially enrolled in the study. Patients who (1) were found to have distant metastasis on the initial staging work-up and received palliative treatment, (2) were diagnosed with ductal carcinoma in situ, (3) had received any type of treatment before staging FDG PET/CT, (4) had received adjuvant 3D-CRT, but, did not undergo post-RT FDG PET/CT, and (5) had previous history of another malignant disease or heart disease such as coronary artery disease and heart failure were excluded. Of the initially enrolled 106 patients, three were excluded, because their FDG PET/CT images could not be analyzed due to image storage problems. Therefore, 103 patients comprised the final study population.

### 2.2. Diagnostic Work-Up and Follow-Up Courses

**Figure 1:**
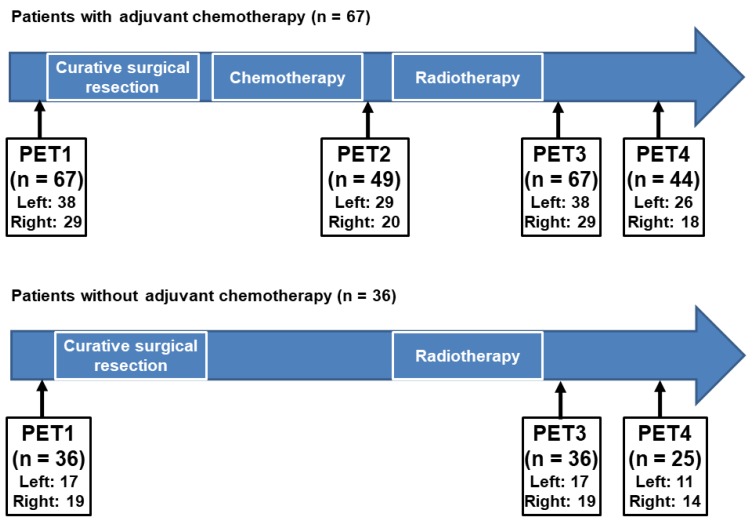
Clinical follow-up course of the enrolled patients. Among the enrolled patients, 67 who had received adjuvant chemotherapy underwent four sequential F-18 Fluorodeoxyglucose (FDG) positron emission tomography/computed tomography (PET/CT)cans that consisted of PET/CT for staging work-up (PET1), PET/CT after the completion of the adjuvant chemotherapy (PET2), PET/CT after the completion of the adjuvant radiotherapy (PET3), and follow-up PET/CT for surveillance (PET4). For 36 patients who did not receive adjuvant chemotherapy, three sequential FDG PET/CT were performed that consisted of PET1, PET3, and PET4. (Left, left breast cancer; Right, right breast cancer).

The diagnostic and follow-up courses of the enrolled patients are shown in Figure 1. Staging FDG PET/CT (PET1), adjuvant RT simulation CT, and post-RT PET/CT (PET3) images were available for all enrolled patients. In addition to PET1, patients had undergone staging examinations including blood tests, bone scintigraphy, breast ultrasonography, and magnetic resonance imaging. Based on the results of the staging examinations, all patients underwent curative surgical resection of the breast cancer lesion and histopathologic staging was assessed according to the 7th Edition of the American Joint Committee on Cancer staging system. Furthermore, the status of the estrogen receptor, progesterone receptor, and human epidermal growth factor receptor 2 and Ki67 expression were determined based on the histopathologic evaluation.

After curative surgical resection, all patients underwent adjuvant RT with/without chemotherapy and/or hormone treatment. Of all patients, 67 (65.0%) received initial adjuvant chemotherapy followed by RT with/without hormone treatment (Figure 1). Of the 67 patients who received both chemotherapy and RT, 49 (73.1%) underwent FDG PET/CT after the completion of the adjuvant chemotherapy (PET2) and all 67 underwent PET3. During clinical follow-up after the completion of adjuvant chemotherapy and RT, 44 patients (65.7%) underwent follow-up FDG PET/CT for surveillance (PET4). Of all patients, 36 (35.0%) received adjuvant RT without chemotherapy (Figure 1). Therefore, none of these 36 patients underwent PET2 scanning, and all underwent PET3. During clinical follow-up, 25 of those 36 patients (69.4%) underwent PET4. The interval between PET1 and curative surgical resection was within 14 days (median, 5 days). The median intervals between the end of chemotherapy and PET2, between the end of RT and PET3, and between the end of RT and PET4 were 13 days (range, 5–36 days), 132 days (78–201 days), and 353 days (266–470 days), respectively.

### 2.3. FDG PET/CT

All FDG PET/CT images from PET1 to PET4 were obtained using a single dedicated PET/CT scanner (Biograph mCT 128 scanner, Siemens Healthcare, Knoxville, TN, USA). The blood glucose levels of patients were measured after at least 6 h of fasting before FDG PET/CT. Approximately 4.07 MBq of FDG was intravenously injected only in patients with blood glucose level lower than 150 mg/dL. After a 60 min uptake period, PET/CT scanning was performed from the skull base to the proximal thigh in the supine position. Initially, non-contrast-enhanced CT scanning for attenuation correction and anatomical information was performed at 100 mA and 120 kV_p_ with slices at a thickness of 5 mm. Afterwards, PET scanning was performed at 1.5 min per bed position using the three-dimensional acquisition mode. PET images were reconstructed using point-spread-function modeling with time-of-flight reconstruction with attenuation correction.

### 2.4. Adjuvant RT

For the RT simulation process, contrast-enhanced spiral CT scanning using the Philips Brilliance Big Bore (Philips Medical Systems, Madison, WI, USA) was performed with 5 mm thickness and a free-breathing protocol. The patients lay supine on a 15 degree angle tilting board (CIVCO C-Qual, Coralville, IA, USA) and abducted both arms above the head for appropriate exposure of their breast.

For adjuvant whole breast RT, planning was performed using 3D-CRT with standard tangential fields using optimal parallel opposed tangential angles. The standard tangential fields were defined as follows: (1) The superior border was the sternal notch or 1 cm above the breast tissue, (2) the inferior border was 2 cm below the inframammary fold, (3) the medial border was the mid-sternum, (4) the lateral border was 2 cm laterally to breast tissue (mid-axillary line). Each angle had two fields with x- and y-axis physical wedges, respectively, for better dose homogeneity. For boost RT to the tumor bed, the clinical target volume (CTV) was defined as the tumor bed including surgical clips, incision scar, and seroma plus a 10–15 mm margin dependent on the surgical margin status. The planning target volume for boost RT was defined as the CTV plus a 10 mm margin for photon boost or a 20 mm margin for electron boost. The prescribed dose of the whole breast was 50.4 Gy in 28 fractions, and the boost dose to the tumor bed was 10–16 Gy in 5–8 fractions. All plans were performed with maximum hot spots lower than or equal to 107% of the prescribed dose.

### 2.5. Image Analysis

All simulation CT and FDG PET/CT data sets were transferred to a radiation treatment planning system (Eclipse ver. 8.9, Varian Medical Systems, Palo Alto, CA, USA). The whole myocardium structure of all patients was contoured on contrast-enhanced simulation CT images. Myocardium contouring was performed by including the left and right ventricles and interventricular septum based on the cardiac contouring atlas for RT [26]. All 3D-CRT plans for the whole breast were reproduced using the same treatment planning condition and energy of each patient. Based on these plans, the 30 Gy and 47.5 Gy isodose areas of the myocardium were converted to a 3D structure. The CT images of PET/CT were merged with simulation CT images to obtain the myocardial FDG parameters. In the primary alignment, the CT images of PET/CT were shifted and tilted to match the simulation CT images with reference to the sternum and ipsilateral ribs. In the secondary fine alignment, a radiation oncologist manually assessed and elaborately modified the CT images of PET/CT based on the location, shape, and angle of the heart.

**Figure 2:**
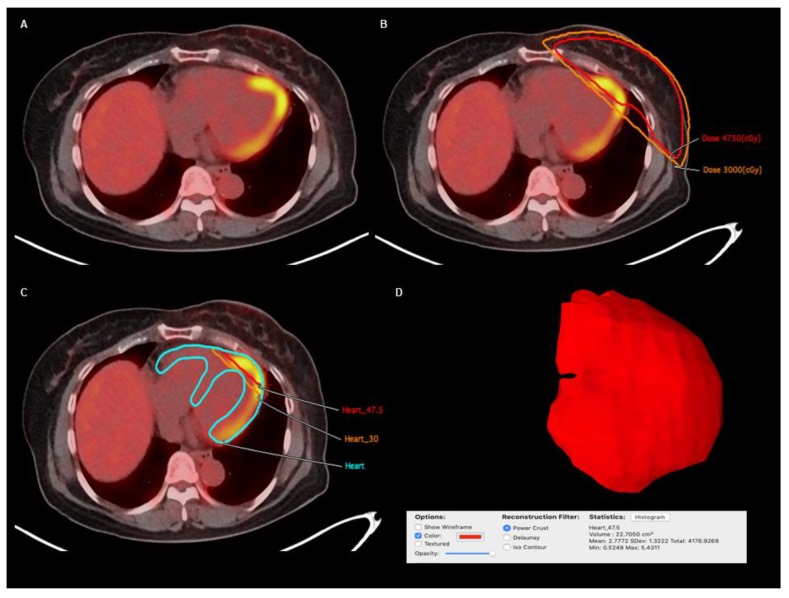
(**A**) Transaxial image and (**B**) transaxial image with dose-distribution on post-radiotherapy F-18 Fluorodeoxyglucose (FDG) positron emission tomography/computed tomography (PET/CT) in a 51-year-old woman with left breast cancer. (**C**) The structures of the whole myocardium (Heart) and myocardium irradiated with 30 Gy (Heart_30) and with 47.5 Gy (Heart_47.5) constructed from CT-stimulation images for radiotherapy were fused with the FDG PET/CT images. For each myocardial structure, the three-dimensional volume structure was automatically constructed and the mean FDG uptake of the volume structure was measured. (**D**) An example of three-dimensional volume structure of myocardium irradiated with 47.5 Gy in this patient is shown, displaying a mean standardized uptake value of 2.7772 for myocardium irradiated with 47.5 Gy.

The structures of the entire myocardium and the irradiated areas of the myocardium were copied from simulation CT images and transferred to each FDG PET/CT image. The FDG uptake of these structures was measured using OsiriX MD (Pixmeo, Geneva, Switzerland) (Figure 2). For both the left and right breast cancer groups, myocardium-to-blood pool uptake ratios were measured to represent the FDG uptake of the whole myocardium. The myocardium on FDG PET/CT images was outlined by the fused myocardium structure derived from the RT simulation CT images and PET/CT images, and the mean FDG uptake of myocardium, expressed as the standardized uptake value (SUV), was calculated. A spheroid-shaped volume-of-interest was drawn over the descending aorta, and the mean SUV of the blood pool was measured. The myocardium-to-blood pool uptake ratio was measured using the mean SUV of the myocardium and of the blood pool.

For patients with left breast cancer, the mean SUVs of the irradiated myocardium were additionally measured along with those of the whole myocardium (Figure 2). Based on the 3D-CRT plans, the whole myocardium was segmented into three sections; the myocardium irradiated with less than 30 Gy or with out of RT field (low-irradiated myocardium), the myocardium irradiated with more than 30 Gy (30 Gy-irradiated myocardium), and the myocardium irradiated with more than 47.5 Gy (47.5 Gy-irradiated myocardium). A threshold radiation dose for the image analysis was determined as 30 Gy, which is considered as the threshold dose for irreversible histologic damage to the myocardium [24,27]. The mean SUV of each segment was measured, and the FDG uptake ratios of 30 Gy-irradiated myocardium-to-low-irradiated myocardium (30 Gy uptake ratio) and 47.5 Gy-irradiated myocardium-to-low-irradiated myocardium (47.5 Gy uptake ratio) were calculated. Furthermore, using the 30 Gy and 47.5 Gy uptake ratios on each PET scan, the percent changes in the 30 Gy and 47.5 Gy uptake ratios between the follow-up PET scans (PET2, PET3, and PET4) and the PET1 scan were calculated as follows: (percent change of the uptake ratio) = ((uptake ratio on follow-up PET scan) − (uptake ratio on PET1))/(uptake ratio on PET1) × 100%.

### 2.6. Statistical Analysis

The statistical analyses of the present study comprised three steps. Firstly, the myocardium-to-blood pool uptake ratios on PET scans between patients with left and right breast cancers and among PET1, PET2, PET3, and PET4 were compared to examine the changes in FDG uptake of the whole myocardium. Secondly, the 30 Gy uptake ratios and 47.5 uptake among between PET1, PET2, PET3, and PET4 in patients with left breast cancer were compared to evaluate the changes in FDG uptake in the irradiated myocardium after 3D-CRT. Finally, differences between the 30 Gy and 47.5 uptake ratios and between percent changes in the 30 Gy and 47.5 Gy uptake ratios on PET scans were assessed to examine whether changes in myocardial FDG uptake were related to the radiation dose to the myocardium. Additionally, we also evaluated whether the clinical risk factors of cardiovascular disease, including hypertension, diabetes mellitus, and dyslipidemia medication, had an influence on the changes of FDG uptake of the myocardium. Student’s *t* test, paired *t* test, and one-way repeated measures analysis of variance (ANOVA) with *post-hoc* pairwise multiple comparisons were performed to compare differences in continuous variables. Bonferroni correction was applied to adjust for multiple comparisons. For categorical variables, the chi-square test and Fisher’s exact test were used to compare the differences in ratios. All statistical tests were two-sided and performed using MedCalc statistical software version 19.1 (MedCalc Software bvba, Ostend, Belgium). A *p*-value of <0.05 was considered statistically significant.

## 3. Results

### 3.1. Patient Characteristics

The 103 enrolled patients comprised 55 with left breast cancer and 48 with right breast cancer, and all underwent both PET1 and PET3 scans. There were no significant differences in clinico-histopathological factors between patients with left and right breast cancers (*p* > 0.05; Table 1). At the time of the staging work-up, two patients with left breast cancer (3.6%) and three patients with right breast cancer (6.3%) had been taking dyslipidemia medications including statin. There were no significant differences in the intervals from treatment to PET scanning between patients with left and right breast cancers (*p* > 0.05; Table 1). Because only a small number of patients had undergone all PET/CT scans from PET1 to PET4 (19 patients (34.5%) with left breast cancer and 14 patients (29.2%) with right breast cancer), differences in variables were evaluated among PET1, PET2, and PET3 and among PET1, PET3, and PET4, instead of evaluating differences among PET1, PET2, PET3, and PET4.

### 3.2. Comparison of Myocardial FDG Uptake between Left and Right Breast Cancers

**Figure 3:**
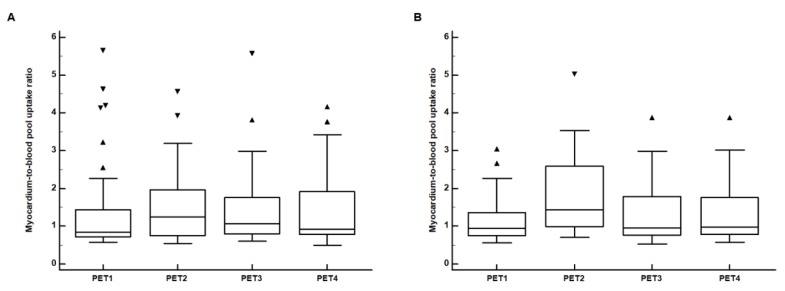
Distributions of the myocardium-to-blood pool uptake ratios on F-18 Fluorodeoxyglucose (FDG) positron emission tomography/computed tomography (PET/CT) for staging work-up (PET1), PET/CT after the completion of the adjuvant chemotherapy (PET2), PET/CT after the completion of the adjuvant radiotherapy (PET3), and follow-up PET/CT for surveillance (PET4) in (**A**) patients with left breast cancer and (**B**) patients with right breast cancer. (Central box, the values from the 25 percentile to 75 percentile; middle line in the box, median value; error bar, extending from the minimum value to the maximum value except outside values; marker, an outside value which is larger than the 75 percentile value plus 1.5 times the interquartile range).

The myocardium-to-blood pool uptake ratios were measured in all patients, and there was no significant difference in the volume of myocardium between patients with left (154.5 ± 27.20 cm^3^) and right (153.3 ± 25.6 cm^3^) breast cancers (*p* = 0.682). No significant differences in the myocardium-to-blood pool uptake ratios between patients with left and right breast cancers were found in any PET scans (*p* > 0.05; Table 2). The myocardium-to-blood pool uptake ratios on PET2, PET3, and PET4 tended to show slightly higher values than that on PET1 in both patient groups. However, in pairwise comparisons, the myocardium-to-blood pool uptake ratio on PET3 showed no significant difference from that on PET1 in both patients with left and right breast cancers (*p* > 0.05; Figure 3). Furthermore, on repeated measures ANOVA, there were no significant differences in the myocardium-to-blood pool uptake ratios among PET1, PET2, and PET3 and among PET1, PET3, and PET4 in both patient groups (*p* > 0.05 for all; Figure 3).

We also evaluated the differences of myocardium-to-blood pool uptake ratios among PET scans according to the history of hypertension, diabetes mellitus, and dyslipidemia medication. On PET1, patients with diabetes mellitus (0.93 ± 0.46) showed significant lower myocardium-to-blood pool uptake ratio than other patients (1.32 ± 0.95; *p* = 0.014), while there were no significant differences of myocardium-to-blood pool uptake ratios on PET2, PET3, and PET4 scans between patients with diabetes mellitus and other patients (*p* > 0.05). Furthermore, no significant differences of myocardium-to-blood pool uptake ratio were shown among PET1, PET2, PET3, and PET4 scans according to the history of hypertension and dyslipidemia medication (*p* > 0.05).

### 3.3. Comparison of the Irradiated Myocardium-to-Non-Irradiated Myocardium Uptake Ratios between PET Scans

The mean SUVs of 30 Gy-irradiated myocardium and low-irradiated myocardium were measured in all 55 patients with left breast cancer. In contrast, the mean SUV of 47.5 Gy-irradiated myocardium was measured only in 47 patients, because the volumes of the 47.5 Gy-irradiated myocardium were too low (lower than 1.0 cm^3^) in eight patients, which might render the result vulnerable to the partial volume effect. The mean volumes of the myocardium irradiated with 30 Gy and 47.5 Gy and of the low-irradiated myocardium were 21.96 ± 12.64 cm^3^, 12.77 ± 8.41 cm^3^, and 122.53 ± 28.63 cm^3^, respectively. The values of the 30 Gy and 47.5 Gy uptake ratios and the percent changes in the 30 Gy and 47.5 Gy uptake ratios are shown in Table 3.

**Figure 4:**
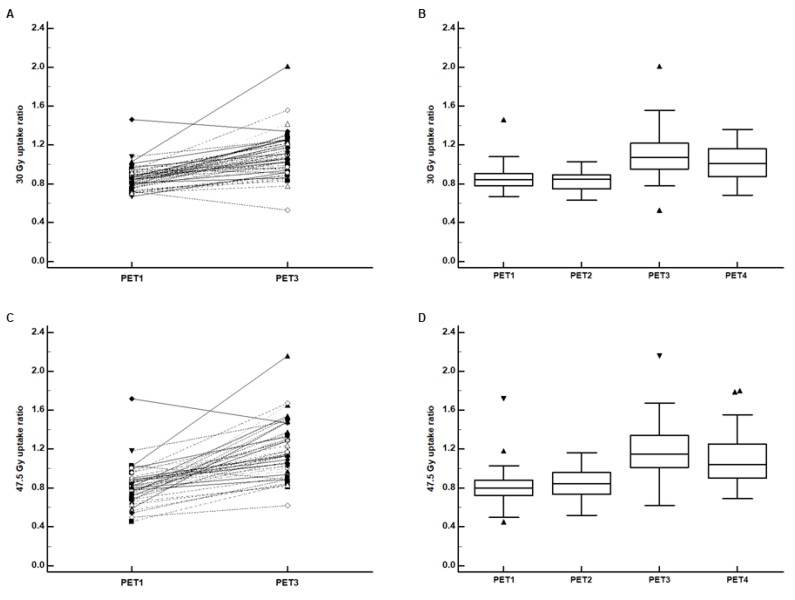
(**A**) Pairwise comparison of the 30 Gy-irradiated myocardium-to-low-irradiated myocardium F-18 Fluorodeoxyglucose (FDG) uptake ratio (30 Gy uptake ratio) between positron emission tomography/computed tomography (PET/CT) for staging work-up (PET1) and PET/CT after the completion of the adjuvant radiotherapy (PET3) and (**B**) distributions of the 30 Gy uptake ratio on PET1 (*n* = 55), PET/CT after the completion of the adjuvant chemotherapy (PET2) (*n* = 29), PET3 (*n* = 55), and follow-up PET/CT for surveillance (PET4) (*n* = 37). (**C**) Pairwise comparison of the 47.5 Gy-irradiated myocardium-to-low-irradiated myocardium FDG uptake ratio (47.5 Gy uptake ratio) between PET1 and PET3 and (**D**) distributions of the 47.5 Gy uptake ratio on PET1 (*n* = 47), PET2 (*n* = 24), PET3 (*n* = 47), and PET4 (*n* = 30). (Central box, the values from the 25 percentile to 75 percentile; middle line in the box, median value; error bar, extending from the minimum value to the maximum value except outside values; marker, an outside value which is smaller than 25 percentile minus 1.5 times the interquartile range or larger than the 75 percentile value plus 1.5 times the interquartile range).

Significantly increased FDG uptake was observed after 3D-CRT in the 30 Gy-irradiated myocardium. In the pairwise comparison between PET1 and PET3, the 30 Gy uptake ratio on PET3 was significantly higher than that on PET1 (*p* < 0.001; Figure 4A), and 51 patients (92.7%) showed higher 30 Gy uptake ratios on PET3 than on PET1. On repeated measures ANOVA, there were significant differences in the 30 Gy uptake ratio among PET1, PET2, and PET3 and among PET1, PET3, and PET4 (*p* < 0.001 for all; Figure 4B). On post-hoc pairwise comparisons between PET1, PET2, and PET3 with Bonferroni correction, the 30 Gy uptake ratio on PET3 was significantly higher than that on PET1 (*p* < 0.001) and PET 2 (*p* < 0.001), whereas there was no significant difference between PET1 and PET2 (*p* > 0.999). On *post-hoc* comparisons between PET1, PET3, and PET4, the 30 Gy uptake ratio significantly decreased on PET4 as compared with PET3 (*p* = 0.022). However, it was still higher than the uptake ratio on PET1 (*p* < 0.001).

The 47.5 Gy-irradiated myocardium also showed significantly increased FDG uptake after 3D-CRT at a more striking degree. In the pairwise comparison, the 47.5 Gy uptake ratio on PET3 was significantly higher than that on PET1 (*p* < 0.001; Figure 4C), and 95.7% of patients (45 of 47 patients) showed an increased uptake ratio on PET3. On repeated measures ANOVA, there were significant differences in the 47.5 Gy uptake ratio among PET1, PET2, and PET3 and among PET1, PET3, and PET4 (*p* < 0.001 for all; Figure 4D). On post-hoc pairwise comparisons between PET1, PET2, and PET3, the 47.5 Gy uptake ratio on PET3 was significantly higher than that on PET1 (*p* < 0.001) and PET 2 (*p* < 0.001), whereas there was no significant difference between PET1 and PET2 (*p* = 0.269). On post-hoc comparisons between PET1, PET3, and PET4, a decreased 47.5 Gy uptake ratio was observed on PET4 compared with PET3, but with borderline significance (*p* = 0.051), and the 47.5 Gy uptake ratio on PET4 was still significantly higher than that on PET1 (*p* < 0.001).

We also investigated the differences of the 30 Gy and 47.5 Gy uptake ratios according to the history of hypertension, diabetes mellitus, and dyslipidemia medication. On all PET scans, there were no significant differences of 30 Gy and 47.5 Gy uptake ratios between patients with and without hypertension, between patients with and without diabetes mellitus, and between patients with and without dyslipidemia medication (*p* > 0.05).

### 3.4. Comparison of FDG Uptake between the 30 Gy and 47.5 Gy Irradiated Myocardium

**Figure 5:**
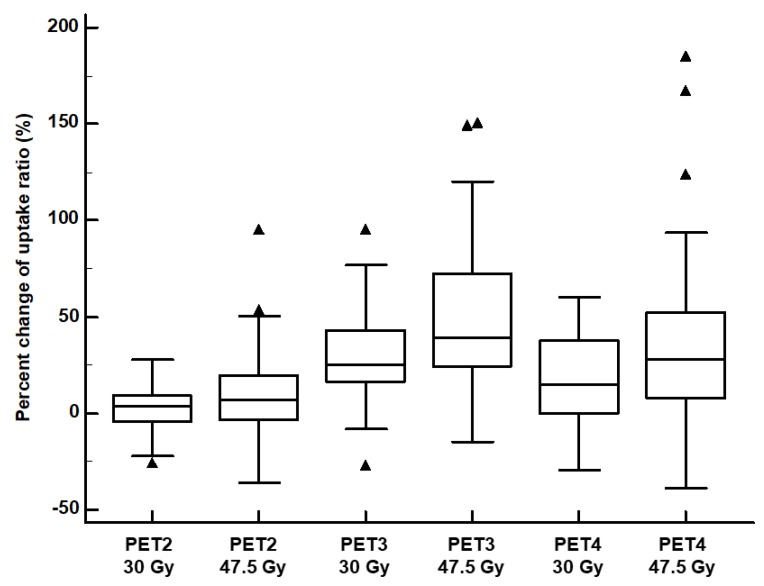
Distributions of percent changes in the 30 Gy-irradiated myocardium-to-low-irradiated myocardium F-18 Fluorodeoxyglucose (FDG) uptake ratio (30 Gy uptake ratio) and 47.5 Gy-irradiated myocardium-to-low-irradiated myocardium FDG uptake ratio (47.5 Gy uptake ratio) on sequential FDG positron emission tomography/computed tomography (PET/CT). (PET2 30 Gy, percent change in the 30 Gy uptake ratio on PET/CT after the completion of the adjuvant chemotherapy; PET2 47.5 Gy, percent change in the 47.5 Gy uptake ratio on PET/CT after the completion of the adjuvant chemotherapy; PET3 30 Gy, percent change in the 30 Gy uptake ratio on PET after the completion of the adjuvant radiotherapy; PET3 47.5 Gy, percent change in the 47.5 Gy uptake ratio on PET after the completion of the adjuvant radiotherapy; PET4 30 Gy, percent change in the 30 Gy uptake ratio on surveillance PET/CT; PET4 47.5 Gy, percent change in the 47.5 Gy uptake ratio on surveillance PET/CT). (Central box, the values from the 25 percentile to 75 percentile; middle line in the box, median value; error bar, extending from the minimum value to the maximum value except outside values; marker, an outside value which is smaller than 25 percentile minus 1.5 times the interquartile range or larger than the 75 percentile value plus 1.5 times the interquartile range).

In pairwise comparisons between the 30 Gy and 47.5 Gy uptake ratios, on PET3 and PET4, the 47.5 Gy uptake ratios were significant higher than the 30 Gy uptake ratios (1.19 ± 0.28 vs. 1.09 ± 0.22; *p* < 0.001 for PET3; 1.10 ± 0.28 vs. 1.01 ± 0.18; *p* = 0.006 for PET4). In contrast, no significant differences were shown between the 47.5 Gy and 30 Gy uptake ratios on PET1 (*p* = 0.235) and PET2 (*p* = 0.713). Furthermore, in pairwise comparisons, on PET3 and PET4, percent changes in the 47.5 Gy uptake ratios were significantly higher than percent changes in the 30 Gy uptake ratios (50.2 ± 36.9% vs. 28.7 ± 22.3%; *p* < 0.001 for PET3; 40.6 ± 51.8% vs. 18.2 ± 23.3%; *p* = 0.002 for PET4; Figure 5). Meanwhile, there was no significant difference between the 47.5 Gy and 30 Gy uptake ratios on PET2 (*p* = 0.104; Figure 5).

## 4. Discussion

In the present study, no significant difference in FDG uptake of the whole myocardium was shown between patients with left and right breast cancers after adjuvant 3D-CRT. However, for patients with left breast cancer, FDG uptake of the myocardium irradiated with more than 30 Gy significantly increased after adjuvant 3D-CRT even on the one-year follow-up PET/CT as well as on the post-RT PET/CT. Furthermore, the degree of FDG uptake increase significantly correlated with radiation dose to the myocardium.

It has been already found that radiation can cause damage to the myocardial microvascular tissue and myocardial metabolism [21,24,28]. In previous studies with animal models, radiation directly affected endothelial capillary cells within the myocardium, resulting in capillary swelling, obstruction of vascular lumens, and perivascular fibrosis [28,29]. These microvascular damages lead to ischemic conditions and fibrotic changes in the myocardium [27,28,29]. Under ischemic conditions, the myocardium switches its main energy source from free fatty acid to glucose and lactate [21,28]. Furthermore, high-dose irradiation to the myocardium also causes degeneration and deregulation of proteins in the myocardial mitochondria, especially for mitochondrial oxidative phosphorylation, pyruvate metabolism, and cytoskeletal structure [28,30,31]. These mitochondrial damages can cause impairment of myocardial oxidative metabolism and a transition to anaerobic metabolism with upregulated glycolysis [20,28,30,31]. Abnormally increased FDG uptake in the irradiated myocardium is known to result from these microvascular and mitochondrial damages [24,28]. In a previous in vivo study with beagles, increased FDG uptake was observed in the myocardium treated with a radiation dose of 20 Gy on PET/CT performed three months after RT and histopathologic evaluation of the heart showed microvascular damage and mitochondrial injury in the irradiated myocardium [28].

In the literature, five clinical studies have evaluated changes in myocardial FDG uptake after RT in esophageal and lung cancer, four studies with 3D-conformal techniques with a conventional radiation dose [21,23,24,25] and one study with the stereotactic body RT technique [20]. Because all of these previous studies were retrospective, only two have compared myocardial FDG uptake between the staging and post-RT PET/CT scans [20,23] and the median intervals between RT and post-RT PET/CT were very diverse, ranging from 25 days to 11 months [20,21,23,24,25]. Except for one study with patients with esophageal cancer, all others consistently showed increased FDG uptake in the irradiated myocardium on post-RT PET/CT images [20,21,24,25]. Similarly, the results of the present study also showed that FDG uptake of the myocardium irradiated with more than 30 Gy significantly increased on post-RT PET/CT in patients with breast cancer who underwent adjuvant 3D-CRT. Moreover, similar to findings of the previous study on esophageal cancer [24], the increase of FDG uptake in the irradiated myocardium was significantly associated with the radiation dose to the myocardium and was persistently observed in the one-year follow-up PET/CT, suggesting that the damage to the myocardium was related to the radiation dose and was not a transitional phenomenon. So far, contradictory results have been reported regarding the direct relationship between myocardial FDG uptake and cardiac toxicity [23,32]. However, considering that the myocardial region of high FDG uptake on post-RT PET/CT revealed decreased perfusion on myocardial perfusion scintigraphy, the increased myocardial FDG uptake on post-RT PET/CT might suggest increased risk of radiation-induced ischemic heart disease [21]. Therefore, the results of our study could be considered imaging evidence that supports a higher risk of heart disease in patients with left breast cancer after adjuvant 3D-CRT [6,7,33].

The most distinct feature of our study is that we used 3D volume-myocardial structures directly constructed from RT simulation CT images for measuring myocardial FDG uptake. By fusing volume structures with PET/CT images, we could measure the mean SUV of the whole myocardium in the cardiac ventricles and the whole area of the myocardium irradiated with 30 Gy or 47.5 Gy, while, because of the difficulty in defining the whole irradiated myocardial areas on PET/CT images, the previous studies mainly used the visual grade or maximum SUV for evaluating myocardial FDG uptake [20,21,23,24,25,32]. The maximum SUV only represents a single voxel value of a volume-of-interest and is susceptible to image noise [34,35]. In contrast, the mean SUV provides information from all voxels of a volume-of-interest; thereby, it is less sensitive to image noise, but is highly dependent on the definition of volume-of-interest [34,35]. As the whole areas of the irradiated myocardium on RT simulation CT were included in our measurement, the mean SUV of the myocardium in our study would more accurately reflect the degree of myocardial FDG uptake than could visual analysis and maximum SUV. Furthermore, to overcome the temporal variability of myocardial FDG uptake [19], we used the 30 Gy and 47.5 Gy uptake ratios, rather than myocardial SUV itself. Using uptake ratios, we were able to compare myocardial FDG uptake between the myocardial regions and between PET/CT scans. In future studies, our measurement method can be used to monitor the changes in myocardial FDG uptake after RT and to compare the degree of radiation-induced myocardial damage between RT techniques.

Cardiotoxic chemotherapeutic agents such as anthracycline and trastuzumab are also known to induce increase of myocardial FDG uptake on post-chemotherapy PET/CT in patients with breast cancer and lymphoma [32,36]. In the present study, the myocardial-to-blood pool uptake ratios on PET2 showed higher values than those on PET1 both in patients with left and with right breast cancer. This finding might be also due to the cardiotoxicity of adjuvant chemotherapy because most of the enrolled patients who had undergone adjuvant chemotherapy had received doxorubicin-based regimens. However, the difference in the myocardial-to-blood pool uptake ratio between PET1 and PET2 failed to reach statistical significance. Considering the various chemotherapeutic regimens used in our study, further studies would be needed to elucidate the impact of chemotherapy on the myocardial FDG uptake in patients with breast cancer.

On previous studies, clinical risk factors for cardiovascular disease including hypertension, diabetes mellitus, and dyslipidemia have been known to be affect FDG uptake of the myocardium [37,38,39]. Therefore, in the present study, we also compared myocardial FDG uptake according to the history of hypertension, diabetes mellitus, and dyslipidemia. The results of our study showed that, on PET1, patients with diabetes mellitus had significantly lower myocardium-to-blood pool uptake ratio than other patients, which has been already shown in the previous study [38]. On the other hand, hypertension, diabetes mellitus, and dyslipidemia medication showed no significant impact on all other comparisons of myocardium-to-blood pool uptake ratio and 30 Gy and 47.5 Gy uptake ratios. These results suggest that the effect of these clinical risk factors to the changes of myocardial FDG uptake after radiotherapy and chemotherapy might be limited. However, because of the small number of patients with hypertension, diabetes mellitus, and dyslipidemia medication in the present study, further study would be needed to investigate the influence of these clinical factors to myocardial FDG uptake after radiotherapy.

The present study had several limitations. First, because the patients in this study were retrospectively selected from a single medical center, selection bias was inevitable. Furthermore, comparisons of FDG PET/CT findings with other cardiac imaging studies could not be performed. Second, because of the relatively short follow-up period, the relationship between the changes in myocardial FDG uptake and cardiac outcome could not be assessed. Third, only a small number of patients had undergone all PET scans from PET1 to PET4. Therefore, we could not perform serial comparisons of all PET scans. Finally, FDG PET/CT images in our study were acquired using an oncologic PET/CT protocol. Given that constituents of diet and fasting duration before PET/CT scanning can affect myocardial FDG uptake [40,41], further prospective studies with controlled dietary regimens and fasting durations are warranted to confirm the changes in myocardial FDG uptake after RT.

## 5. Conclusions

In the present study, significantly increased FDG uptake of the myocardium irradiated with more than 30 Gy was shown on PET/CT after adjuvant 3D-CRT in patients with left breast cancer. The increase of myocardial FDG uptake was significantly associated with radiation dose to the myocardium and was persistently observed in the one-year follow-up PET/CT after 3D-CRT. The changes in myocardial FDG uptake appeared to support the increased risk of heart disease after RT in patients with left breast cancer. Further studies are required to validate the results of the study.

## Figures and Tables

**Table 1:** Characteristics of the enrolled patients.

Characteristics		Patients with Left Breast Cancer (*n* = 55)	Patients with Right Breast Cancer (*n* = 48)	*p*-Value
Age (years)		51.2 ± 9.9 *	51.4 ± 9.7 *	0.928
Menopausal status	Premenopausal	22 (40.0%)	17 (35.4%)	0.634
	Postmenopausal	33 (60.0%)	31 (64.6%)	
Histopathology	Intraductal carcinoma	48 (87.3%)	41 (85.4%)	0.785
	Intralobular carcinoma	7 (12.7%)	7 (14.6%)	
T stage	T1	33 (60.0%)	35 (72.9%)	0.191
	T2	19 (34.5%)	9 (18.8%)	
	T3	3 (5.5%)	4 (8.3%)	
Lymph node metastasis	Absence	41 (74.5%)	34 (70.8%)	0.674
	Presence	14 (25.5%)	14 (29.2%)	
Tumor size (cm)		1.82 ± 1.20 *	2.09 ± 1.13 *	0.252
Histologic grade	Grade 1	17 (30.9%)	15 (31.2%)	0.980
	Grade 2	28 (50.9%)	25 (52.1%)	
	Grade 3	10 (18.2%)	8 (16.7%)	
Estrogen receptor status	Positive	49 (89.1%)	39 (81.2%)	0.263
	Negative	6 (10.9%)	9 (18.8%)	
Progesterone receptor status	Positive	42 (76.4%)	30 (62.5%)	0.128
	Negative	13 (23.6%)	18 (37.5%)	
HER2 status	Positive	27 (49.1%)	23 (47.9%)	0.906
	Negative	28 (50.9%)	25 (52.1%)	
Triple negative tumor		2 (3.6%)	3 (6.2%)	0.662
Ki67 expression status	Positive (≥14%)	29 (52.7%)	27 (56.2%)	0.722
	Negative (<14%)	26 (47.3%)	21 (43.7%)	
Hypertension	Yes	8 (14.5%)	11 (22.9%)	0.277
	No	47 (85.5%)	37 (77.1%)	
Diabetes mellitus	Yes	8 (14.5%)	9 (18.8%)	0.568
	No	47 (85.5%)	39 (81.2%)	
Dyslipidemia medication	Yes	2 (3.6%)	3 (6.3%)	0.540
	No	53 (96.4%)	45 (93.7%)	
Serum total cholesterol level on staging blood tests		187 ± 29 *	194 ± 35 *	0.340
Serum triglyceride level on staging blood tests		143 ± 95 *	134 ± 103 *	0.662
Adjuvant treatment	CTx+RT+Hx	37 (67.3%)	26 (54.2%)	0.322
	RT+Hx	17 (30.9%)	18 (37.5%)	
	CTx+RT	1 (1.8%)	3 (6.2%)	
	RT alone	0 (0.0%)	1 (2.1%)	
Chemotherapy regimen	AC	14 (25.5%)	9 (18.8%)	0.551
	TAC	11 (20.0%)	12 (25.0%)	
	CMF	8 (14.5%)	7 (14.6%)	
	FAC	3 (5.5%)	1 (2.1%)	
	FEC	2 (3.6%)	0 (0.0%)	
Prescribed total radiation dose (Gy)	Whole breast RT dose	Median, 50.4 (range, 50–50.4)	Median, 50.4 (range, 45–50.4)	0.440
	Boost RT dose	Median, 16 (range, 8–16)	Median, 16 (range, 10–16)	0.225
Boost RT location	UOQ	24 (49%)	24 (57.1%)	0.077
	UIQ	11 (22.4%)	10 (23.8%)	
	LOQ	4 (8.2%)	6 (14.3%)	
	LIQ	4 (8.2%)	2 (4.8%)	
	Central	6 (12.2%)	0 (0%)	
Interval between CTx and PET2 (days)		11.9 ± 4.6 *	13.5 ± 6.3 *	0.322
Interval between RT and PET3 (days)		139.7 ± 28.1 *	132.0 ± 28.2 *	0.170
Interval between RT and PET4 (days)		357.4 ± 50.2 *	363.6 ± 49.3 *	0.605

* Expressed as average ± standard deviation. HER2, human epidermal growth factor receptor 2; CTx, chemotherapy; RT, radiotherapy; Hx, hormonal therapy; AC, doxorubicin and cyclophosphamide; TAC, docetaxel, doxorubicin, and cyclophosphamide; CMF, cyclophosphamide, methotrexate, and fluorouracil; FAC, fluorouracil, doxorubicin, and cyclophosphamide; FEC, fluorouracil, epirubicin, and cyclophosphamide; UOQ, upper outer quadrant; UIQ, upper inner quadrant; LOQ, lower outer quadrant; LIQ, lower inner quadrant; PET2, post-chemotherapy positron emission tomography/computed tomography (PET/CT); PET3, post-radiotherapy PET/CT; PET4, surveillance PET/CT after adjuvant treatment.

**Table 2:** The myocardium-to-blood pool uptake ratios in patients with left (*n* = 55) and right (*n* = 48) breast cancers.

Patients	Myocardium-to-Blood Pool Uptake Ratio			
	PET1	PET2	PET3	PET4
Patients with left breast cancer	1.33 ± 1.10	1.54 ± 1.07	1.41 ± 0.92	1.47 ± 1.00
Patients with right breast cancer	1.26 ± 0.59	1.61 ± 1.16	1.47 ± 1.16	1.43 ± 0.89
*p*-values	0.318	0.357	0.695	0.869

All data were expressed as average ± standard deviation. PET1, staging positron emission tomography/computed tomography (PET/CT); PET2, post-chemotherapy PET/CT; PET3, post-radiotherapy PET/CT; PET4, surveillance PET/CT after adjuvant treatment.

**Table 3:** 30 Gy uptake ratios, 47.5 Gy uptake ratios, and the percent changes in the 30 Gy and 47.5 Gy uptake ratios in patients with left breast cancer.

Variables	PET1 (*n* = 55)	PET2 (*n* = 29)	PET3 (*n* = 55)	PET4 (*n* = 37)
30 Gy uptake ratio	0.85 ± 0.12	0.84 ± 0.10	1.09 ± 0.22	1.01 ± 0.18
Percent change in the 30 Gy uptake ratio (%)	-	1.7 ± 11.5	28.7 ± 22.3	18.2 ± 23.3
47.5 Gy uptake ratio	0.81 ± 0.20 *	0.83 ± 0.17 †	1.19 ± 0.28 *	1.10 ± 0.28 ‡
Percent change in the 47.5 Gy uptake ratio (%)	-	7.0 ± 28.7 †	50.2 ± 36.9 *	40.6 ± 51.8 ‡

All data were expressed as average ± standard deviation. * Measured in 47 patients; † Measured in 24 patients; ‡ Measured in 30 patients. 30 Gy uptake ratio, 30 Gy-irradiated myocardium-to-low-irradiated myocardium uptake ratio; 47.5 Gy uptake ratio, 47.5 Gy-irradiated myocardium-to-low-irradiated myocardium uptake ratio; PET1, staging positron emission tomography/computed tomography (PET/CT); PET2, post-chemotherapy PET/CT; PET3, post-radiotherapy PET/CT; PET4, surveillance PET/CT after adjuvant treatment.
